# Impact: a case study examining the closure of a large urban fixed site needle exchange in Canada

**DOI:** 10.1186/1477-7517-7-11

**Published:** 2010-05-25

**Authors:** Joan MacNeil, Bernadette Pauly

**Affiliations:** 1 School of Nursing, University of Victoria, P.O. Box 1700, Victoria, BC, V8W 2Y2, Canada

## Abstract

**Introduction:**

In 2008, one of the oldest fixed site needle exchanges in a large urban city in Canada was closed due to community pressure. This service had been in existence for over 20 years.

**Case Description:**

This case study focuses on the consequences of the switch to mobile needle exchange services immediately after the closure and examines the impact of the closure on changes in risk behavior related to drug use, needle distribution and access to services The context surrounding the closure was also examined.

**Discussion and Evaluation:**

After the closure of the fixed site exchange, access to needle exchange services decreased as evidenced by the sharp decline in numbers of clients reached, and the numbers of needles distributed and collected monthly. Reports related to needle reuse and selling of syringes suggest changes in risk behaviors. Thousands of needles remain unaccounted for in the community. To date, a new fixed site has not been found.

**Conclusion:**

Closing the fixed site needle exchange had an adverse effect on already vulnerable clients and reduced access to comprehensive harm reduction services. While official public policy supports a fixed site, politicization of the issue has meant a significant setback for harm reduction with reduced potential to meet public health targets related to reducing the spread of blood borne diseases. This situation is unacceptable from a public health perspective.

## Introduction

Needle exchange programs have been operating with government funding throughout Canada since the late 1980s. They are generally regarded as one of the most important factors in preventing HIV epidemics among those who use injection drugs in Canada [1,2]. Most community-based needle exchange programs provide a nonjudgmental setting for people who use injecting drugs to dispose of used injection equipment, access sterile syringes and other injecting paraphernalia, condoms and HIV prevention education [3]. Many also offer free HIV testing, counseling and support and referrals for health and other social services. Community based needle exchanges were developed over two decades ago in response to concerns about risks of injection-related HIV transmission [4,5].

On May 31, 2008, after over twenty years of operation, the single fixed site needle exchange in one large urban center closed its doors. The purpose of this paper is to describe events associated with the closure of the only fixed site in the city and to examine the impact of the closure on patterns of risk behavior related to drug use, distribution of injection supplies and access to services.

The overarching research question was "what was the impact of the closure on drug related risk behavior, needle distribution and access to servicesγ" We are focusing specifically on the perspectives of clients and providers as to the impact of the closure on patterns of drug use in the community, risk behaviors associated with drug use, the numbers of needles exchanged pre and post closure, and changes in access to services.

## Case Description

Case study methodology is used most effectively to study specific phenomena in a real world context when it is not possible to separate the phenomena of study from the setting or context [6,7]. Case studies are particularly useful when it is not possible to manipulate variables and it is important to include the contextual factors that are relevant to the phenomena being studied [6]. In this report, the phenomenon of interest is the impact of the closure of the single fixed site needle exchange in a large urban center. Thus, the unit of analysis or case, is the closure of the fixed site needle exchange. We have used a chronological approach and focused on describing the series of events including the historical, social and political factors that contributed to the closure and the aftermath of the closure of the fixed site.

The data included for analysis were drawn from local police call data pre and post closure for the street and surrounding streets where the fixed site was located, agency records related to needle distribution and client contacts pre and post closure, ten in-depth semi-structured interviews with clients, 3 client focus groups (consisting of six, five, and five clients respectively), one focus group consisting of six service providers, five individual interviews with health care providers and police officers and participant observation. Participant observation included observing mobile outreach. During mobile outreach, one of the researchers accompanied street outreach workers on bicycle or foot, and the other researcher accompanied workers at a mobile van.

Data were collected over a five month period after the closure. This length of time was selected to allow clients to adjust to the change in service delivery but also to be recent enough that clients would recall the use of the fixed site. In consultation with the outreach workers, we anticipated that the closure would have impacts on patterns of risk behaviors related to drug use, needle distribution and access to services and wanted to better understand and describe these impacts. We sought to gain an understanding of the phenomena from a variety of perspectives in order to enhance data quality and provide a basis for comparison and contrast in relation to the impact of the closure.

Before data collection began, this study was approved by the researchers' university ethics board, as well as the NGO providing the exchange services. Voluntary informed verbal or written consent was obtained before each interview, observation, and focus group. All of the interviews and focus groups were audiotaped and transcribed. Field notes were recorded and transcribed. Reoccurring themes and patterns related to the impacts of the closure were identified. Findings were checked and confirmed between the researchers and with agency staff and clients.

### Historical, Political and Social Context

Needle exchange services in this city were initiated in 1988 with first a mobile then a single fixed site exchange in the downtown core. Over the years, the NGO- run services expanded to include street outreach, health referrals, sexual and drug education, health promotion, HIV positive support groups and prison outreach. The populations accessing these services also increased from a handful of clients in 1988 to several thousand by 2008 [8].

In 2007, a feasibility study on "supervised consumption options" in the city [9] identified critical service gaps for drug users, specifically with regard to detoxification, treatment, housing/shelter, and basic social and health care. These gaps were also identified seven years earlier in another research study [10]

While the exact number is not known, there are an estimated 1,500 to 2,000 people who use injection drugs in the city area [11,12]. Illicit substance use, including injection drug use, often coincides with homelessness, increasing the vulnerability of individuals to not only HIV and hepatitis C, but also to poor health as a result of inadequate shelter, poor nutrition, violence and poverty. As part of a homeless needs survey, it was estimated that about 1, 242 people in this city were homeless or unstably housed [13]. Although 78% of participants cited affordable housing as a barrier, almost half (47%) of the participants in that survey reported alcohol or drug use and 41% of participants indicated that alcohol and drug use was one of three major factors contributing to inadequate housing. At the time of this case study analysis, another survey of 105 clients of the needle exchange, found that more than half the sample were homeless, had lived in the area for a long time, and were an older street-entrenched population of injection drug users [14].

Despite efforts to reach more clients with comprehensive HIV and hepatitis C (HCV) prevention services, some disquieting trends exist. This city was one site among a total of seven Canadian cities, for the I-Track study, a cross-sectional surveillance survey of risk behaviours and prevalence of HIV and hepatitis C virus (HCV) among people who use injection drugs. The data for the I-Track study (n = 250) revealed that the prevalence of HIV and HCV remains unacceptably high among those who use injecting drugs at 15.4% for HIV and 68.5% for HCV [15]. In addition, drug consumption patterns could be contributing to the increased risk of exposure. While heroin may be injected 1-2 times a day, addiction to cocaine requires more frequent injections. Over 70% of the people who reported using injection drugs in the 2006 sample, reported injecting cocaine as their most common drug over the past 6 months, with over 50% reporting injecting more than 6 times per day [15].

Prior to the closing in 2008, the fixed site needle exchange was open seven days a week from 3 pm to 11 pm in the evening with additional hours on Sundays. Services, including needle exchange, were provided by the outreach staff. Nurses were at the exchange at these times every week day, to provide health services such as abscess care, counseling, testing for STDs, HIV and HCV as well as health referrals. In addition to the needle exchange and nursing services, the fixed site offered addictions referrals, shelter requests, clothing requests, hospital referrals (rides), transportation referrals, phone outreach, counseling, hygiene supplies, comprehensive prevention education, other harm reduction services, and sometimes food.

Despite the increased demand for harm reduction services in the city, the NGO operating the fixed site exchange received an eviction notice from their landlord. This notice was in response to complaints from neighbors on the street regarding open street drug use, loitering and garbage. The landlord offered that if the organization closed the fixed site needle exchange, the NGO could remain in the building and continue delivery of other support and health services which included a large HIV Support program, educational sessions and volunteer outreach. Prior to the closure, the NGO worked with the city and the regional health authority to try to find a new location for a fixed site needle exchange but due to concerns of nearby neighbours, particularly an elementary school, efforts to relocate to a new site were unsuccessful [16-20]. Following the closure, a community based needle exchange advisory committee was mobilized with the goal of finding a new location for a fixed site [21]. After over 18 months, multiple attempts to find a location and final rejection of a selected site by those who use drugs among others, the committee was disbanded [22]. While the city and public health authorities articulate support of harm reduction priorities and services [23-25], more than a year and a half after the closure, a new location for a fixed site has not been identified.

Following the closure of the fixed site, service delivery shifted from a fixed site needle exchange to mobile services. Services consist of a van parked on a side street away from the downtown core area and mobile outreach on bicycles and by foot. The street where the van is parked was agreed upon by the city, the police and the NGO providing the needle exchange services. The van exchange and mobile outreach operate in the evenings, seven days a week. After the closure of the fixed needle exchange, nursing services were available at the NGO building site only one afternoon per week, whereas prior to the closure, the nurses were at the fixed site needle exchange, every evening from 3 pm to 11 pm. Although nurses also increased their strolls and drives around the downtown core, the hours of outreach nursing services were reduced with the closure of the fixed site. Following the closure of the fixed site, efforts were made by other service providers to increase secondary distribution of clean supplies for injecting with prepackaged packets of syringes and injecting equipment prepared and available to clients upon request. The health authority also increased the number of sites where injecting equipment is available [26].

Pressure from neighborhood groups also led to the creation of a "No go Zone" post closure for mobile needle exchange services. This "No go Zone" consisted of the street where the fixed exchange had existed and covered a two block radius where a private elementary school and a large homeless shelter were located. The rules for the "No go Zone" were that harm reduction outreach workers could not conduct any needle exchange in this area. However, this area is frequented by many clients of the needle exchange, especially those who are homeless. As part of the "No go Zone" outreach workers were instructed to ask clients who requested clean supplies to walk with them to an area outside of the zone before they could provide clean supplies for injecting. Also code of conduct prohibited needle exchange in front of residences, open businesses, schools and day-care centers. Outreach staff are expected to abide by this Code of Conduct not to conduct needle exchange in these areas.

### Changes in Distribution of Needles Pre and Post Closure

After the closure of the fixed site, the numbers of needles distributed and collected by the NGO decreased dramatically (see Table 1). Needle distribution in June, 2008, after the closure, was down 40% and intake decreased by 72% compared to April. The numbers of needles distributed and recovered has continued to be lower than pre-closure rates. Previously, the NGO service provider reported a greater than 80% return rate [8]. At the same time, outreach workers are not reporting finding more discarded needles on the streets. Even though some other service providers are distributing and recovering needles, the numbers overall have not reached pre-closure levels (see Table 2). Thus, leaving has thousands of needles unaccounted for in the community, potentially being reused or shared.

**Table 1:** Needles Distributed and Recovered by the NGO in Victoria in 2008 and 2009.

2008	Distributed	Recovered
April	28,038	26,562
May* The closure was May 31.	n/a	n/a
June	16,700	7.500
July	25,000	19,000
August	27,000	10,700
September	24,473	21,647**
October	22,095	7,966
November	24,862	16,503
December	14,885	5,932
January, 2009	16,256	10,139
February, 2009	17,900	18,200
March, 2009	21,100	9,100
April, 2009	21,943	17,361
May, 2009	18,037	8,961
TOTAL	285,151	177,409

*The data base of the NGO corrupted the data for May so the numbers are not available.

** This includes one client who brought in 10,000 needles.

**Table 2:** Needles distributed and recovered by all service providers in Victoria in 2008 and 2009.

2008	Distributed	Recovered
April	45,400	36,900
May* May 31st was the closure		
June	24,700	13,400
July	33,400	24,000
August	36,500	19,700
September	34,213	27, 383* *
October	29,805	12,617
November	34,405	23,914
December	26,494	18,743
January, 2009	25,146	17,140
February, 2009	26,100	27,200
March, 2009	28,000	18,000
April, 2009	33,035	23,064
May, 2009	24,095	19,235
TOTAL	401,293	281,296

* The data base for the NGO corrupted the data for May so the numbers are not available

**Includes one client who returned 10,000 needles.

### Change in Number and Type of Client Contacts

The number of clients accessing the needle exchange dropped dramatically after the closure of the fixed site, from 373 in May to 273 in June and 277 in July. The majority of the clients reached in June and July were reached by the outreach on foot and/or the bikes, as opposed to the mobile van.

After the closure of the fixed site, outreach services were reduced because the range of services  available at the fixed site were  no longer available on outreach. The nature of interactions with clients has changed with contacts becoming of shorter duration. Outreach staff reported that clients literally just take their needles and move on. There is no safe place to sit and talk. This was confirmed by the researchers' observations on outreach. No one lingers at the mobile sites whereas before the closure, at the fixed site, clients could sit down inside off the street, talk with an outreach worker or counselor, visit the nurse in the private clinic room, or sit and have a cup of coffee. One of the outreach workers, made the following observation,

"On outreach, people are actively using. They are with their peers. It is a street culture where there is no confidentiality. The dynamics of interactions on the street are different and we're not able to talk about issues....Being inside away from the craziness of the street creates an opportunity."

Staff noted that at the fixed site they were better able to develop relationships with clients as the site provided a safe place to meet, talk and develop trust. The site also served as a communication hub where people could find out what had happened to friends, use the phone to call family, receive calls from clients in treatment or in jail, and importantly, to find out about any dangerous drugs on the street and what to avoid. Information obtained from clients and staff confirmed that the fixed site offered a consistent and readily accessible place and service for people with little consistency in their lives. The needle exchange was viewed by clients as a safe haven from the street that provided a trusted point of access to services [27].

### Changes in Displacement of Clients and Drug Use

In the month after the closure, clients expressed fear and anxiety, in part related to the loss of the fixed site and the increased police presence. People on the street indicated that they were trying to keep out of the public eye and not wanting to or able to linger in any one spot. In many interviews and discussions, clients, workers and others indicated that there are many people whom they had not seen since the closure. This was confirmed by the outreach workers, who noted that people were harder to find. Other health care providers also reported that even though they increased their strolls and driving around the downtown core, they were seeing fewer clients and receiving fewer telephone calls.

The following quotes from three individual in-depth client interviews illustrate the effect of these pressures and the displacement of people making it difficult to locate clients.

"People have been going out of their way to try to get out of the public's eye so that we can be out of the way"

"People are under pressure-no safe place to go. People are moved on and harassed. A guy was picking up pop cans at the XXXX and was charged with public loitering (by the police)."

"Everybody's lost, everyone's scattered all over the place, there's not one set spot. People are scattering all over the place."

As noted above, several clients reported that the closure of the needle exchange meant a loss of a safe place to go.

The police call data for streets or areas where focus groups and outreach workers had noted an increase in clients after the closure of the fixed site, were reviewed. Police call data, confirmed that police calls for loitering and disturbances in all the neighboring streets after the closure increased dramatically. For example, police calls for drugs and unwanted persons on a street two blocks from the former fixed site needle exchange jumped from 19 in 2007 to 55 in 2008, with the increases coming after the closure of the fixed site. This interview and police call data appear to support the findings from the three focus groups with clients and from the ten individual in-depth interviews, that people and drug use have moved further afield. The key implication is that closure of the fixed site needle exchange led to a spreading out of drug use into adjacent areas and further afield into other areas of the community. 

### Changes in Access to Services

As outlined above, the hours of service for needle exchange and access to nursing services and other housing and social services have been reduced as a result of the closure of the exchange. During the 10 in-depth interviews, all clients reported increased difficulties and less access to services as a result of the closure of the fixed site as the quotes below demonstrate:

"Oh, I sure liked it a lot better when it was in a fixed site. Yeah, of course it's great that we can get new needles...but it is really hard cause my HIV has affected my nerves and it is hard for me to walk."

"It's not that far....And not only that. I don't know where they are half of the time. Not like at the needle exchange."

"Only accessed them a couple times because usually I cannot find them"

A sense of fear prevailed in the initial reaction to the outreach workers on bikes and the mobile needle exchange. Three clients reported that they initially thought that the outreach workers were police on bikes and that the parked van was a police van with a camera. This was confirmed as well, in one of the 3 focus groups with clients. Three months after the closure, it appeared that some of these fears had decreased as clients became more familiar with the van and the outreach workers on their bikes, but contacts were still sporadic and very short. In addition, continued pressure from police to break up groups and move people on was reported by clients as "constantly being under pressure". This pressure left many clients feeling vulnerable and harder to find as this client noted below:

"..today the staff that were on bikes came to see us because they were wondering where to find people because of the cops kicking us out of other places. We cannot have one specific place so it is hard for them to find us. If they can't find us, they can't give us clean things to use."

The inability to locate clients and the feeling of being constantly moved on is akin to pushing drug use underground with the potential for increased risk behaviors and lack of access to clean injection supplies as shown by this last quote above.

The clients who had accessed the services at the fixed site before the closure, and were now users of the mobile services, all stated in their individual in-depth interviews that the bike outreach was good and something that should be kept, but that it did not replace a fixed site. This is demonstrated by the following client quotes:

"They need to have a needle exchange, a permanent one...You know, just like the old one. Where we could go for coffee and talk, you know. At least needles weren't spread all over the place."

"I know a lot of people who were clean and sober, not using, they went there and sat in the back part of the place to get away from everything else...it was like a socially positive environment for them to stay off drugs by going there. Which doesn't sound...it's weird but that is what was going on..."

### Changes in Risk Behaviors Related to Drug Use

Most of the clients interviewed were accessing the outreach services for needles, water, condoms and sometimes for food. In response to the question "When you need new clean needles can you get themγ" two of the clients said "yes, always", but the others said "no, not always". One client said "*because it is too hard for me to figure out where they are going. But now I know about this mobile van. But all those times I had no idea where to get them. It's just more complicated now". *Another said he accessed the mobile services as follows "*only a couple of times a month because I usually cannot find them. Yeah, but I've got lots of friends who usually get boxes of syringes that I can just go and see them."*

Others commented that the van was too far away from where they stayed downtown, and two made reference to buying needles on the street as demonstrated in this response "*Yes, usually someone has them. If not, if there is no one here, it might cost you*...." Another client responded "*Do you think someone is going to walk all the way across town to find out they are not even here for a needle....they're going to find the easiest way. *Another client noted the change as follows: *"Used to be able to. And so most people are willing to share the rigs that they have. Most people are turning a dollar for a clean rig. Well, not going around selling, but if you ask them for one, they ask you for a buck." *Informal discussions on outreach affirmed that the price of buying syringes increases in the evening and overnight with one estimate that a syringe would cost about $5.00 at midnight.

In response to the question "*The last time you injected, did you use a clean needleγ" *eight clients reported reusing their own needles. This was also confirmed by the outreach workers, "*People are reusing their own syringes. I have clients tell me that they reuse their needles five to six times." *Other clients, during interviews and the focus groups, reported that they had seen people picking up dirty needles and using them. This contrasts with with over thirty clients interviewed at the other needle exchanges outside of the city who reported that they could always access a clean needle and that they never reused their own needles [27]. One client summarized succinctly, the impact of the closure when he stated:

"Now we have decreased access to health care, decreased access to support services, health, education, counseling or referrals."

Outreach workers reported that over 70% of clients who asked them for safe supplies in the "No-service Zone" were not willing to walk the two blocks and instead, change their minds about needing clean supplies. Reasons for not wanting to walk the two blocks include lack of mobility, concern about their leaving their possessions behind and an unwillingness to give up their spot outside the homeless shelter. It may be that when clients were asked to meet workers outside of the 'No service Zone", this was viewed as one more request to move on and a feeling that workers were having to police "No service Zones" with the potential for erosion of trust that is so essential to the delivery of harm reduction services.

## Discussion and Evaluation

The provincial government of this Canadian province has a policy on harm reduction that states that community partners will work to provide a full range of harm reduction services that include, but are not limited to referrals, advocacy, education and supplies distribution, and that these services must respect their clients by adhering to basic ethical principles [28]. This policy and the provincial harm reduction guidelines [29] cite, as a community example, the efforts this city took in 2004 to embrace harm reduction as a pragmatic, cost effective and socially responsible approach to reducing the personal and social harms associated with substance use. In spite of these policy positions, the fixed needle exchange was closed as a result of public pressure.

In responding to a description of the struggle in Vancouver to establish a safer injecting facility, Wodak [30] noted that "all drug politics is local" (p.83). He stated that cities are now more important than states or nations in the process of transition from criminal justice dominated approaches to harm reduction reforms. This could certainly be said for the closure of the fixed site where local politics and public disorder, rather than public health dominated, resulting in the closure of the longstanding fixed site.

A number of studies have shown that limitations or disruptions to syringe distribution coverage -- in combination with a variety of other local environmental factors -- may have an adverse effect regarding levels of risk behavrior and potentially also, HIV or HCV transmission [31-34]. In Australia, in 1999, a needle exchange was closed in a city with a large Aboriginal population, reportedly because of press reports and photographs showing non-indigenous youth injecting close to the site where injecting equipment was distributed by the exchange. At the time it was predicted that this closure could lead to a future HIV epidemic among former clients [35]. Another closure of a needle syringe exchange program in northwestern Sydney in 2002 occurred following negative local media attention and led to a recommendation that media provide balanced and accurate reporting of drug use [36].

In the United States, the Windham, Connecticut needle exchange closed after becoming embroiled in a public controversy in which it was blamed for the city's drug problem, discarded syringes, and even the economic decline of the city itself. Follow-up after the closure revealed significant increase in the percentage of respondents who reported an unreliable source as their primary source of syringes, in respondents' reports of the frequency of reusing syringes, and in the percentage of respondents who reported sharing of syringes, resulting in the city's drug injectors engaging in higher levels of risk behavior [37]. This is similar to the findings of this case study.

Models for the delivery of needle exchange services have been described including peer outreach, mobile services, fixed sites and secondary distribution. Strike et al.[38], in an ethnographic study of Ontario's needle exchanges, described the use of four models for delivery of needle exchange services including fixed sites, mobile services, home visits and satellite sites. They found that fixed and mobile sites reach different groups of people, and that both are needed. Fixed sites have the advantage of providing more confidential spaces for counseling and increased referrals. Mobile services tend to reach higher risk users who may not otherwise access services but provide less confidential spaces. Certainly, the response from clients interviewed for this study, after the closure, would support both fixed and mobile services.

A year and a half after the closure of the fixed site needle exchange, not much has changed. The search continues for a suitable location for a fixed site, the numbers of clients contacted and needles exchanged remain low, and informal reports of difficulties reaching clients and accessing clean injection equipment continue. This is despite supportive editorials in the newspapers and journalists' articles lamenting the situation [39,40] Resources were not available to conduct follow-up interviews, or to do pre- and post closure serological testing. However, as the days and months continue to pass without a fixed site needle exchange for people who use injecting drugs in the city, an already extremely vulnerable population continues to be at increased risk of transmission of blood borne infections and abcesses, and suffers from a lack of access to health care and social services.

### Strengths and Limitations

There are number of benefits associated with the use of case study research methodology [6]. In particular, the benefits of case study research are enhanced through the use of multiple sources of evidence, creation of a case study database and maintaining a chain of evidence. In this study, we drew on documents, police data, interviews and focus groups with outreach workers, clients, health care providers and police as well as study observations. Considerable data was available because the closure of the needle exchange was a high profile public issue of significant public interest. Case studies are generally strengthened by the inclusion of alternative perspectives on the phenomena under study [6]. In this case study, we mainly drew on the perspectives of clients and providers as well as publicly available documents that provided historical, social political context related to the closure as our intent was to look at the impact for these groups. Thus, we did not seek out alternative perspectives on the closure such as that of the neighbours next to the needle exchange pre-closure or the school or businesses located in proximity to proposed sites. Further, the findings of single descriptive case studies such as this, cannot be generalized to other settings although this study does provide beginning insight into potential impacts from a client and provider perspective.

## Conclusion

The closure of one of Canada's busiest and oldest fixed site needle exchange services and the switch to mobile delivery only has had a traumatic effect on clients, with reported increases in risk behavior such as needle reuse as well as a dramatic decrease in access to services. Contacts with vulnerable clients have been lost and thousands of needles are unaccounted for in the community. Outreach staff continue to reach out to clients on the street but lament the loss of contact with many former clients and the loss of a comprehensive harm reduction approach to services. This is a set back for harm reduction that is unacceptable from a policy perspective as well as from a social justice perspective. One of the basic tenets of harm reduction is the right to comprehensive, non-judgemental medical and social services and the fulfillment of basic needs for all individuals and communities affected by drug use. Although this article did not directly address or analyze the political and social factors affecting the closure, the current situation appears to be primarily an outcome of the interplay of those factors in spite of scientific evidence and official policies.

## Competing interests

The authors declare that they have no competing interests.

## Authors' contributions

JM, BP initiated the study and performed the analysis. JM prepared the first draft. BP provided input into the manuscript. All authors approved the final manuscript for publication.
